# Parenting style and children emotion management skills among Chinese children aged 3–6: the chain mediation effect of self-control and peer interactions

**DOI:** 10.3389/fpsyg.2023.1231920

**Published:** 2023-09-18

**Authors:** Dexian Li, Wencan Li, Xingchen Zhu

**Affiliations:** ^1^School of Education, Liaoning Normal University, Dalian, China; ^2^College of Psychology, Liaoning Normal University, Dalian, China

**Keywords:** authoritarian parenting style, authoritative parenting style, emotion management skills, self-control, peer interactions, chain mediating effects, Chinese children

## Abstract

Drawing on ecosystem theory, which is based on the interaction of family environment, individual characteristics, and social adaptation, this study aimed to examine the effects of parenting style on emotion management skills and the mediating roles of self-control and peer interactions among Chinese children aged 3–6 years. Some studies have investigated the relationship between parenting style and emotion management skills. However, research on the underlying mechanisms is still deficient. A sample of 2,303 Chinese children completed the PSDQ-Short Version, the Self-Control Teacher Rating Questionnaire, the Peer Interaction Skills Scale, and the Emotion Management Skills Questionnaire. The results show that: (1) Authoritarian parenting style negatively predicted children’s emotion management skills, self-control, and peer interactions; (2) Authoritative parenting style positively predicted children’s emotion management skills, self-control, and peer interactions; (3) Structural equation models indicated that self-control and peer interactions partially mediated the effects of authoritarian and authoritative parenting styles. The parenting style of Chinese children aged 3–6 years is related to emotion management skills, and self-control and peer interactions have chain mediating effects between parenting style and children’s emotion management skills. These results provide further guidance for the prevention and intervention of emotional and mental health problems in children.

## Introduction

Behavioral and emotional difficulties commonly occur during childhood, often without recognition or awareness, yet they have a profound impact on mental well-being and behavioral patterns. Early and middle childhood encompass a broad spectrum of behavioral issues in children, including defiance, tantrums, aggression, and destructive tendencies (American Psychiatric Association, 2013). In the late 20th century, concerns arose regarding the rising prevalence of emotional and behavioral problems among children and adolescents, leading to an interest in addressing and modifying these secular trends (Rutter and Smith, 1995). Recent nationally representative data from the NSCH reveals the widespread occurrence of depression, anxiety, and conduct problems among children and adolescents in the United States (Ghandour et al., 2019). Based on a report released by the National Center on Birth Defects and Developmental Disabilities from 2005 to 2011, it is approximated that around 13–20% of children in the United States face a mental, emotional, or behavioral disorder each year (Avenevoli et al., 2013). Increased levels of aggression have been associated with emotional instability (Carlo et al., 2011; Mestre et al., 2012), as well as addictive behaviors, conduct issues, and borderline personality (Mehroof and Griffiths, 2010). However, a preschooler’s ability to conform to societal behavioral standards is influenced by both emotional comprehension and regulation (Di Maggio et al., 2017). Generally, possessing strong emotional management skills indicates adaptability and good health, while inadequate management can contribute to individual social maladjustment and behavioral problems. Neglecting to address these issues leads to considerable societal expenses and places a substantial burden on individuals and communities (Scott et al., 2001; Romeo et al., 2006), underscoring the importance of timely intervention.

Individual variations in emotional tendencies may serve as an implicit underlying factor influencing the diverse externalizing behaviors observed in children. However, the factors that shape children’s ability to regulate their emotions are intricate and multifaceted. Parenting styles within the familial context exert a substantial direct or indirect impact on children’s emotional and mental well-being, receiving considerable attention in the field of child growth and development both domestically and internationally (Ong et al., 2018; Feng et al., 2021; Rongeven et al., 2022). Numerous studies have explored the association between childhood anxiety disorders (Sahithya and Raman, 2021), emotional well-being (Le and Impett, 2019), oppositional defiant disorder (Fooladvand et al., 2021), and parenting styles. Additionally, research has investigated the efficacy of the Emotional Development (ED) module of Parent–Child Interaction Therapy (PCIT) in treating early childhood depression (Luby et al., 2020). However, few studies have delved into the intricate mechanisms through which various parenting styles influence children’s emotion regulation skills, exploring the complex and intriguing network of relationships involved. Spanish researchers approached psychological research on children aged 4–6 years from the sociocultural perspective of psychological development theory, proposing the Morris triple model of early childhood adjustment (Badenes et al., 2000), which serves as the theoretical foundation for this study. The model comprises three components: (a) family factors influencing individual emotions, (b) the impact of young children’s social adjustment on individual emotions to varying degrees, and (c) the influence of family factors on young children’s adjustment. In this study, parenting style represents the family aspect, children’s emotion regulation skills represent the individual emotion aspect, and factors influencing young children’s social adjustment encompass their self-control abilities (Wang et al., 2019) and peer interactions (Badenes et al., 2000; Domberg et al., 2018). Consequently, parenting styles not only directly affect children’s emotional well-being but also exhibit lasting effects on their psychological characteristics and the development of peer relationships. The development of self-control and peer interactions has been shown to be significantly influenced by family factors, particularly parenting styles and behaviors (Russell et al., 2013; Wang et al., 2019; Ahmetoglu et al., 2022). Therefore, investigating the mechanisms through which parenting style influences children’s emotion regulation skills is essential. The objective of this study was to examine the impact of parenting style on children’s emotion regulation skills and the mediating roles played by self-control and peer interactions.

### The effect of parenting style on emotion management skills

The family plays a pivotal role in a child’s growth, yet other influential environments, including schools (Chao, 2001; Veiga et al., 2021) and peer groups (Maccoby and Martin, 1983; Martinez-Escudero et al., 2023), also contribute to their development. Both parental involvement theory and bioecological theory indicate that parenting style significantly influences the healthy development of children’s emotion management skills (Morris et al., 2007; Luo et al., 2019). Parenting styles encompass a range of parental attitudes and behaviors toward raising children (Darling and Steinberg, 1993), reflecting the quality of children’s interactions with family members and their emotional well-being. Parenting approaches can be characterized by integrating two core dimensions: responsiveness and demandingness (Lamborn et al., 1991; Villarejo et al., 2023). These dimensions, namely responsiveness and demandingness, are typically seen as independent of each other (Darling and Steinberg, 1993; Fuentes et al., 2022). The term “responsiveness” captures a parent’s warmth, active involvement, and ability to nurture a child’s unique personality (Baumrind, 1991; Climent-Galarza et al., 2022). On the other hand, “demandingness” describes the level of strictness and expectations parents set for their children in aligning with societal or familial norms (Steinberg et al., 1994; Palacios et al., 2022). Within this dual-dimensional framework, several scholars have outlined specific parenting categories. These include authoritative (high in both responsiveness and demandingness), authoritarian (high demandingness, low responsiveness), neglectful (low in both dimensions), and indulgent (high responsiveness, low demandingness) as common classifications in familial research (Lamborn et al., 1991; Steinberg et al., 1994; Villarejo et al., 2023). Baumrind’s work (Baumrind, 1968; Baumrind, 1971) has distinguished between permissive-indulgent and permissive-neglectful parents, noting both a variance in levels of parental responsiveness and resultant child outcomes (Lamborn et al., 1991; Martinez-Escudero et al., 2020). Interestingly, children from families emphasizing responsiveness but lacking in demandingness often exhibit better adaptability and skills compared to their counterparts from neglectful households. Building on Baumrind’s foundational theoretical approach, Maccoby and Martin (1983) advanced the classification of parenting styles into four distinct categories: authoritative, authoritarian, indulgent, and neglectful. Interestingly, parenting styles and their impacts vary across cultures (Pinquart and Kauser, 2018; Martinez-Escudero et al., 2023). The cultural backdrop can significantly influence the dynamics between parenting methods and child adjustment (Pinquart and Kauser, 2018; Garcia et al., 2019). Considering China’s rich cultural tapestry deeply rooted in Confucian principles, parenting often mirrors its hierarchies and moral teachings. Such teachings promote understanding and respecting familial hierarchies and social statuses. Chinese societal norms value a child’s proper upbringing, which emphasizes elements of guidance and discipline. Therefore, parents may employ stern disciplinary measures if children fall short of set expectations, viewing this strictness as their responsibility (Chao, 1994). This rigorous parenting instills in the young an understanding of their roles within family and society, facilitating their smooth societal integration (Pan and Shang, 2023). In studies of European-American families, an authoritative approach frequently emerges as optimal for child development (Maccoby and Martin, 1983; Lamborn et al., 1991; Steinberg et al., 1994). However, China’s competitive social fabric has intensified parental involvement in children’s upbringing. There’s an evident trend of parents being increasingly directive yet responsive to their children’s needs, with neglectful parenting being a rarity (Henry et al., 2020; Liu F. et al., 2020; Liu J. et al., 2020; Vogel et al., 2021). Contemporary young Chinese parents are veering toward promoting children’s independence, challenging the traditional norm of grandparents’ indulgence. This change has led to a decline in overly permissive parenting practices (Scharff, 2022; Lin et al., 2023; Guo J. et al., 2023). Research by Wu (1996) suggests that Chinese parents frequently resort to reprimands to guide their children’s behavior, particularly in academic realms, reflecting an authoritarian approach (Kriger and Kroes, 1972; Lin and Fu, 1990). Interestingly, recent studies from European and South American contexts highlight the potential advantages of high responsiveness combined with low demandingness (Gimenez-Serrano et al., 2022; Martinez-Escudero et al., 2023; Villarejo et al., 2023). Historically, China has emphasized social harmony and order. Consequently, in Chinese familial contexts, most parents not only respond to their children’s expressions and actions but also set expectations for their emotional and behavioral conduct. An authoritative approach resonates with many Chinese parents, aligning with the cultural ethos of balance. In general, several studies focusing on Chinese-American families have identified the benefits of the authoritative style (Chao, 1994, 2001). Therefore, it can be argued that the two primary parenting styles, authoritative (characterized by both responsiveness and demandingness) and authoritarian (characterized by responsiveness without demandingness), are most prevalent in Chinese families. This research aimed to determine which of these two styles is optimal.

Researchers have long studied the mechanisms through which parenting styles influence children’s emotional health (Li J. B. et al., 2019; Li Y. et al., 2019). This research complements and builds upon Gottman’s classification of parenting styles related to emotion (Gottman et al., 1996). However, studies have typically reported differential manifestations of children’s emotional dysregulation (Camisasca et al., 2022), emotional monitoring and behavioral problems (Haslam et al., 2020) under different parenting styles, or the profound impact of children’s perceived parenting style on their mental health (Feng et al., 2021). Nevertheless, little in-depth research has been conducted on the complete system of children’s emotion management skills. According to family systems theory (Bowen, 1993), the degree of individual socialization determines how individuals experience emotional states, and at the same time, the degree of individual emotionalization is closely related to personal, family, and cultural contexts (Lewis, 1995). Thus, the family has been described as the primary environment for children’s emotional establishment and social adjustment patterns. Research has shown that authoritarian parenting styles (e.g., discipline, control, say no) negatively affect children’s emotional and psychological stability (Kawabata et al., 2011). Additionally, in Helping Families Change, Satir et al. (1994) suggest the importance of family communication theory, which states that 93% of emotional states in parent–child conversations are perceived by each other, and that an emotionally unstable or impulsive child who elicits a hostile parental response may make the child’s response more aggressive than the child’s response prior to the parent’s response (Wahl and Metzner, 2012). In contrast, authoritative parents employ positive emotional communication, characterized by active listening, understanding, and positive recognition, along with consistent rules for their children, which positively influences their children’s emotional management and psychological adjustment (Khaleque, 2013). Family systems theory highlights the critical impact of overall family system stability, harmony, and health on children’s emotional and psychological development (Lindahl et al., 2012). While numerous theoretical and empirical studies have demonstrated the influence of parenting styles on children’s emotion management skills, the role of protective factors has been relatively understudied. Firstly, research on self-control in school-aged children suggests that parent-grandparent co-parenting can predict children’s self-control through maternal authoritative parenting, and authoritative parenting itself is associated with children’s self-control (Yang et al., 2023). Pratt and Cullen (2000) meta-analysis have unequivocally shown that low self-control is significantly and positively correlated with involvement in crime and deviant behaviors. Additionally, Gibbs et al. (1998) found that low self-control mediated a substantial portion of the effect of parenting on college deviance, encompassing behaviors such as alcohol use, class-cutting, academic dishonesty, and school suspension or expulsion. Secondly, punishment and harsh parenting styles can have adverse effects on children, which can be magnified within peer interactions, leading to disharmony in social relationships (Shen et al., 2015). The effects of these variables may manifest not only in the short term but also during long-term developmental processes. In fact, detrimental effects on peer relationships, such as becoming targets of exclusion and bullying, can arise from characteristics of the parent–child relationship, including abusive, neglectful, or maladaptive parenting (Reijntjes et al., 2010, 2011; Lereya et al., 2013). Both children’s self-control and peer interactions exert powerful influences on their emotion management skills. Studies have indicated that individuals with higher levels of peer interactions and self-control tend to hold more positive beliefs about managing negative emotions and expressing positive emotions (Chow et al., 2015; Meng et al., 2020). Consequently, we hypothesize that both self-control and peer interactions mediate the relationship between parenting style and children’s emotion management skills. We proposed that parenting style is related to children’s emotion management skills (hypothesis one). We expect that authoritative parenting will have a positive correlation with children’s emotion management skills, while authoritarian parenting will have a negative correlation.

### The mediating effect of self-control between parenting style and emotion management skills

The ability of children to consciously regulate their behavior in order to meet their specific needs contributes to enhanced focus, improved social adjustment, and effective coping with negative emotional experiences such as pain and frustration (Chen et al., 2012). Self-control refers to an individual’s capacity to regulate behavior, emotions, and various other responses in a timely manner to accomplish specific goals and serves as a significant indicator of early socialization (Moffitt et al., 2011). It encompasses attentional aspects, such as the ability to shift or sustain attention, as well as behavioral aspects, such as inhibiting impulses or undesirable behaviors (Duckworth and Kern, 2011). The initiation hypothesis of the psychological mechanisms of self-control (Mischel and Ayduk, 2002) suggests that children’s attention, cognition, and emotions mature as they develop self-control, with higher levels of self-control reducing an individual’s arousal in emotionally charged situations and regulating emotional expression and impulsive behaviors. Prior studies have established a significant correlation between self-control and prosocial behavior among children and adolescents, indicating that individuals with higher levels of self-control are more likely to exhibit prosocial acts (Carlo et al., 2012). Children experiencing challenges in self-control are susceptible to emotional and behavioral issues (Chui and Chan, 2015). Inadequate regulation of negative emotions can contribute to chronic emotional distress, leading to elevated levels of anxiety and depression (Nigra et al., 2015), ultimately manifesting as irritable moods and pathological behaviors commonly associated with autism. Therefore, adequate self-control facilitates individuals in planning their actions, managing their emotions, conserving ego-depleting resources, and effectively anticipating conflict precursors, making it easier to maintain emotional stability in the face of arbitrary outcomes (Hofmann et al., 2009).

Furthermore, the development of self-control is significantly influenced by family dynamics, particularly parenting styles (Russell et al., 2013). Authoritarian parenting styles, characterized by frequent negative emotions and caregiver disengagement, can hinder children’s adaptive efforts to regulate their behavior and impede the cultivation of self-control (Harrist and Waugh, 2002; Li et al., 2015). Parental rejection and excessive intervention have been found to predict children’s low self-control, as well as heightened levels of anger, anxiety, and aggression (Wang et al., 2015). The imposition of high levels of parental control and intervention may suppress children’s intrinsic motivation, subsequently limiting their exploration and adaptation abilities (Guan et al., 2018). Additionally, research suggests that individuals with higher levels of self-control demonstrate better regulation of mood and emotions, whereas poor self-control is associated with anxiety and depression (Özdemir et al., 2014; Yen et al., 2014; Pan et al., 2021). Given that poor self-control serves as a risk factor for mood disorders, it may contribute to comorbidity between anxiety-depression, substance use, and Internet addiction during later childhood (Na et al., 2017). Some empirical studies have shown that authoritative parenting facilitates children’s perceptions of behavioral autonomy, so that children believe they can make and stick to their own decisions (Yu et al., 2013), have a better sense of control and accomplishment over themselves, and reinforce their positive behaviors. Children with high levels of proprioceptive self-control have fewer externalizing problems and less dissociative distress, and are more socially competent with skills to control negative emotions, get along with others, and follow social rules (Spinrad et al., 2007; Sun et al., 2022). Calm thinking and stable emotion management allow them to focus their energy and time on solving practical problems, prefer a positive view of things, and have an optimistic personality (Baesu, 2019; Li J. B. et al., 2019; Li Y. et al., 2019). The presence of positive interactions, coupled with authoritative parenting, is linked to enhanced self-control outcomes in children throughout their developmental stages. For instance, children who engage in more mutually positive and prosocial interactions with their caregivers exhibit improved behavioral and emotional regulation (Kim and Kochanska, 2012; Davis et al., 2017). Therefore, based on the aforementioned research, we hypothesized that self-control could mediate the effects of parenting style on children’s emotion management skills (hypothesis two).

### The mediating effect of peer interactions between parenting style and emotion management skills

Group dynamics theory suggests that as children grow older, the proportion of peer group participation in children’s lives will increase substantially, and that interactions in different social situations will satisfy children’s intrinsic values (Lewin, 1948; Burnes and Bargal, 2017). Peer acceptance is closely related to emotional perception and positive personality (Beazidou and Botsoglou, 2016). Peer interactions is the comprehensive ability of young children to perceive, adapt, coordinate, and manage peer relationships during interactions with peers of the same or similar age (Jiang et al., 2015). Children’s peer interactions play an important role in children’s emotion management skills, the better children’s peer interactions are, the more they promote children’s ability to identify their own and others’ emotions, manage their bad emotions, and use positive emotions to make better decisions (Mayer et al., 2004). Children who are unpopular, rejected, and ostracized by their peers often develop inappropriate emotional expressions and behavioral conflicts, but inappropriate emotions and behaviors can be observed, learned, imitated, and corrected through peer interactions to reshape emotional perceptions and have stable emotion management. When children’s ability to recognize expressions and select emotion perspectives is better developed, they are more likely to be accepted by their peers (Denham and Couchoud, 1990). Therefore, children’s peer interactions are one of the necessary conditions to determine their level of emotion management skills.

The tendency to exhibit behavioral deficits or excessive behavior increases with poorer peer interactions, and the lack of social skills leading to peer alienation predisposes to the development of autism spectrum disorder (ASD), which interferes with normal interpersonal interactions in adulthood (Corbett et al., 2014). Social learning theory states that children tend to develop their own patterns of interpersonal interactions by observing their parents’ everyday interactive behaviors (Bandura, 1976; Ladd and Kochenderfer-Ladd, 2019). Safdar and Zahrah (2016) found that children raised by authoritative parents were more dominant in peer interactions, more likely to integrate into groups and be accepted by peers, and had a significant positive correlation with peer cooperation behaviors (Lau and Power, 2020). Children with authoritarian parenting, on the other hand, are overly dependent on parents, have less self-control, are more likely to exhibit disobedient behavior, have poorer peer interactions, and often suffer from peer rejection (Lee and Wong, 2009). This situation can increase children’s pride or low self-esteem, and even lead to school bullying, social withdrawal, and self-blocking behaviors, resulting in negative emotional experiences and negative social relationship evaluations (Zheng et al., 2019).

In a study conducted in Spain, the examination of parenting style and peer attachment as predictors of emotional instability in late childhood revealed that both parenting style and peer group played equally significant roles in predicting emotional instability among children (Llorca-Mestre et al., 2017). The findings align with the principles of ecological theory (Bronfenbrenner, 1979), which emphasize the continuous interaction between individual characteristics and their context. According to this theory, individual characteristics evolve and develop over time through ongoing interactions with their environment (Bronfenbrenner and Ceci, 1994). Therefore, when children’s emotional needs are not met by their parents, contextual interactions among peers play a compensatory role in children’s emotional management. Children may be able to learn from their peers how to control their emotions, suppress unreasonable desires, coordinate with others, and rationally handle peer conflicts (Wang et al., 2019). When children can correctly understand their peers’ basic mental states, such as thoughts, intentions, and emotions, and can predict and interpret their peers’ behavior through emotional transference, they will have normal, friendly interactions with their peers and perform better on tasks (Astington and Barriault, 2001). Children who are unpopular, rejected, and ostracized by their peers are more likely to experience bullying (Zhu et al., 2020) and may become more sensitive to frustration, which can further exacerbate levels of problem behavior and contribute to impaired interactions and poor social adjustment in adolescence (Buhs and Ladd, 2001). These negative childhood experiences can further increase a child’s withdrawal behaviors and rejection of peer interactions, resulting in a withdrawn personality disorder (Guina, 2016; Bang et al., 2018). There is a strong correlation between children’s emotion management and well-being and parenting styles and children’s peer interactions (Malonda et al., 2019; Zhang and Deng, 2022). Therefore, we hypothesized that parenting styles may be predictors of peer interactions, such that authoritarian parenting styles negatively predict peer interactions, whereas authoritative parenting styles positively predict peer interactions. Based on the aforementioned research, we hypothesized that peer interactions would mediate the effects of parenting style on children’s emotion management skills (hypothesis three).

### The mediating effect of self-control and peer interactions between parenting style and emotion management skills

Existing research has demonstrated the protective influence of both self-control and peer interactions on the development of children’s emotion management skills (Li et al., 2013; Xu et al., 2021). Moreover, parenting style indirectly affects children’s emotion management skills through the mediating factors of self-control and peer interactions, forming a chain mediating process within the relationship (Erkan and Sop, 2018; Farzadi et al., 2021). Consequently, there is a crucial need for comprehensive investigations into the mechanisms through which parenting styles impact children’s emotion management skills. However, limited studies have directly explored the interplay between self-control, peer interactions, and their mediating effects on the association between parenting styles and children’s emotion management skills. Therefore, this study aims to examine the relationship among these four variables by drawing upon relevant theoretical frameworks and empirical evidence.

Multiple research studies have consistently demonstrated that children’s ability to exercise self-control contributes positively to their social adjustment and interactions with peers. Drawing from a situational-developmental theory perspective, children’s self-control plays a significant role in shaping their preferences for peers, thereby influencing the development of emotional stability in later stages (Llorca-Mestre et al., 2017). Children with high levels of self-control exhibit better regulation of their behavior and emotions, leading to more appropriate and positive interactions with peers. Consequently, favorable group preferences contribute to higher levels of peer status and act as a protective factor against internalization problems in children (Heinrich and Gullone, 2006). In contrast, children with low self-control tend to interpret social cues in group settings as threatening or hostile toward themselves, which can trigger intense emotional reactions and a hostile disposition, making them more prone to engaging in bullying behaviors (Plexousakis et al., 2019; Camodeca and Nava, 2022). It is evident that high levels of self-control are associated with increased opportunities for social interactions, which, in turn, impact children’s ability to engage with peers, form social relationships, enhance their sense of belonging, and foster positive emotional experiences during peer interactions. Additionally, Chinese parents place a strong emphasis on fostering their children’s socialization, including the control of negative emotions and the suppression of desires to prioritize group interests and attain a favorable position within peer relationships (Liu et al., 2017). Given the link between children’s self-control and their peer interactions—both of which jointly and adaptively moderate and manage their emotions and behaviors—this study proposes a third pathway. We speculate that parenting styles impact children’s emotion regulation skills through a chain mediation process that involves both self-control and peer interactions (hypothesis four).

## Materials and methods

### Participants

A total of 2,397 participants were recruited from 16 kindergartens (both public and private) in 10 provinces in seven geographic regions (northern, eastern, southern, central, northwestern, southwestern, and northeastern) of China using stratified cluster sampling. Data collection took place at the start of the spring school year in 2023. Prior permission was obtained from the schools, and written informed consent was obtained from both parents and teachers of the participants. The informed consent forms ensured anonymity and confidentiality and did not require signatures or include subject names. Parents and teachers were clearly informed that participation in the study was voluntary, and they had the right to withdraw at any time. Those who agreed to participate independently completed the paper-and-pencil questionnaire during the parent-teacher conference, with the guidance of a trained research assistant. After completion, the questionnaires provided by the children’s parents and teachers were sealed in envelopes. A small token of appreciation, such as a flower or snack, was given to participants as a gesture of gratitude. Upon screening, 94 questionnaires were identified as invalid due to significant missing information. Thus, a total of 2,303 questionnaires were included in the final analysis, resulting in an overall response rate of 96.08%. Participants were aged 3–6 years (*M* = 4.00, SD = 0.499), 1,180 (51.2%) were boys and 1,123 (48.8%) were girls, 1,082 (34.0%) were in junior class, 782 (34.0%) in middle class, 737 (32.0%) in senior class. The two questionnaires were completed by 476 fathers and 1,827 mothers. These participants were categorized into three age groups (*M* = 31.00, SD = 0.532): 229 were under 30 years of age, 1,702 were between 30 and 40 years of age, and 372 were over 40 years of age.

Before the survey was started, the local education department and the survey schools were consulted to obtain their approval to conduct the study. During the survey, we asked the principal and teachers about the basic conditions of the campus, and the researcher informed the parents and teachers about the purpose and procedures of the study and administered the questionnaires. The questionnaires were administered in the classroom, where teachers conducted parent-teacher conferences with parents. Participants provided written consent before completing the study questionnaire and were informed of the purpose, confidentiality, and anonymity of this study.

All study materials in this study were reviewed and approved by the research ethics committee of the corresponding author’s university.

### Instruments

#### Parenting styles and dimensions questionnaire-short version

The PSDQ-Short Version (Robinson et al., 2001) with 27 items is a modified version of the original 62-item PSDQ (Robinson et al., 1996), developed to measure parent’s self-own behavior and report their behavior with children. The shortened version of the PSDQ consists of two dimensions: authoritarian parenting style and authoritative parenting style, and was completed by the parents. An example item is “I punish my children by excluding them, without explanation”. Each item based on a 5-point Likert-type scale ranging from 1 (Never) to 5 (Always). The scale has been shown to have good reliability and validity. Also, it has shown excellent psychometric properties in the Chinese subject population (Robinson et al., 2001; Li et al., 2012). In this study, the Cronbach’s alpha coefficient of the subscales of authoritarian parenting style and authoritative parenting style were 0.95 and 0.92, respectively.

#### Self-control teacher rating questionnaire

The 22-item Self-Control Teacher Rating Questionnaire (SCTRQ) (Dong, 2005) is a modified version of the original 33-item Self-Control Rating Scale (SCRS-C) by Kendall and Wilcox (Kendall and Wilcox, 1979). The SCRS was developed to assess children’s levels of self-control and is evaluated by their teachers. An example item is “The child can stop what he or she is doing to listen when the teacher starts talking.” Items in the questionnaire are scored on a five-point scale. There are questions with reverse scoring (questions 17 and 22), and the questionnaire encompasses a total of four dimensions. Evidence has been provided in studies of the effects of parenting practices and self-control on problem behavior in left-behind and non-left-behind children in rural China (Zhang et al., 2023). The Cronbach’s alpha coefficient of the scale in this study was 0.94.

#### Peer interaction skills scale

The Peer Interaction Skills Scale (PISS) (Zhang, 2002; Qin, 2022; Qin and Caizhen, 2022) was designed to assess children’s peer interaction skills. It encompasses four dimensions: social initiative, verbal and non-verbal interactions, social barriers, and pro-social behavior. Vandell (2000) noted that teachers can influence children’s peer interaction skills through their own perceptions of the classroom. Therefore, the questionnaire was completed by the teachers based on the children’s daily learning and living performance. A sample item reads, “The child can proactively introduce himself/herself to new peers.” The PISS contains four dimensions measured on a four-point scale. The higher the total score, the higher the level of peer interaction skills. In this study, the Cronbach’s alpha of this scale was 0.84.

#### Emotion management skills questionnaire

The 30-item Emotion Management Skills Questionnaire (EMSQ) (Wu, 2013; Qiu et al., 2021) comprises three dimensions and is completed by teachers, who assess children’s daily emotional expressions and reactions. The questionnaire contains 11 items related to emotion perception skills, 13 items focused on emotion regulation skills, and 6 items assessing emotion utilization skills. Emotion perception refers to the child’s ability to accurately identify both their own and others’ emotional states, as well as to perform preliminary analyses to express emotions appropriately (e.g., “The child notices when the moods of friends around them change”). Emotion regulation pertains to the child’s ability to manage their emotions in accordance with organizational norms specific to a given situation (e.g., “The child takes the initiative to stop a fight between peers”). Emotion utilization skills involve the child’s capability to convey emotions appropriately through facial expressions and behavior to foster positive interpersonal relationships (e.g., “When they are happy or sad, they do not display it overtly and tend to appear emotionally reserved”). Each item is measured using a 5-point Likert scale to evaluate the child’s ability to manage their emotions effectively. The higher the score of children’s emotional management skills scale，the better the children’s ability to maintain their own emotional stability. In this study, the Cronbach’s alpha coefficient of the subscale in the present study was 0.94.

### Data analysis

Statistical analysis in this study was conducted using SPSS 26.0 and Mplus 8.3. First, we used Harman’s one-way method to test for the presence of common method bias (Zhou and Long, 2004). Second, we completed descriptive statistics and Pearson correlation analysis on all study variables by running SPSS 26.0. Considering that gender, age, and grade level may be additional influencing factors for children aged 3–6 years, we decided to control for these variables in the analysis. Third, structural equation modeling (SEM) was used to examine mediating effects. To explore the mediating effects of children’s self-control and their peer interactions in the pathway of parenting styles on children’s emotion management skills, we conducted variable-by-variable analyses of authoritative and authoritarian parenting styles to assess a diverse set of hypotheses. Finally, the bootstrap method (Tibshirani and Efron, 1993) was used to repeat the sample 5,000 times to test the mediating effect of children’s self-control and peer interactions in the pathway of parenting style on children’s emotion management skills.

## Results

### Common method bias test

Harman’s one-way method was used to test for the existence of common method bias (Zhou and Long, 2004). Exploratory factor analysis resulted in 12 factors with eigenvalues greater than 1. The first factor accounted for 36.90% of the total variance, which is less than 40% of the critical standard, indicating that common method bias is not apparent. Therefore, data analysis can proceed.

### Descriptive statistics and significance test

Bivariate correlations between the core variables were performed using Pearson correlation analysis. The results are presented in Table 1, including the means, SDs, and Pearson correlation coefficients of the core variables. The results indicated that all the core variables were significantly related. Emotion management skills were negatively associated with authoritarian parenting style (*r* = −0.640, *p* < 0.001), but positively associated with authoritative parenting style (*r* = 0.733, *p* < 0.001), self-control (*r* = 0.723, *p* < 0.001), and peer interactions (*r* = 0.700, *p* < 0.001). Authoritarian parenting style was negatively associated with self-control (*r* = −0.656, *p* < 0.001) and peer interactions (*r* = −0.570, *p* < 0.001). Whereas authoritative parenting style was positively associated with self-control (*r* = 0.586, *p* < 0.001) and peer interactions (*r* = 0.602, *p* < 0.001). In addition, self-control was positively associated with peer interactions (*r* = 0.663, *p* < 0.001).

**Table 1:** Descriptive statistics and correlation analysis of all the variable.

	1	2	3	4	5
1. Authoritarian parenting style	–				
2. Authoritative parenting style	−0.584***	–			
3. Self-control	−0.656***	0.586***	–		
4. Peer interactions	−0.570***	0.602***	0.663***	–	
5. Emotion management skills	−0.640***	0.733***	0.723***	0.700***	–
Mean	2.925	3.889	3.529	2.717	3.747
SD	0.476	0.760	0.958	0.407	0.618

*p < 0.05, **p < 0.01, ***p < 0.001.

### Total effect, direct effect, and indirect effect of the chain mediating effect

**Figure 1:**
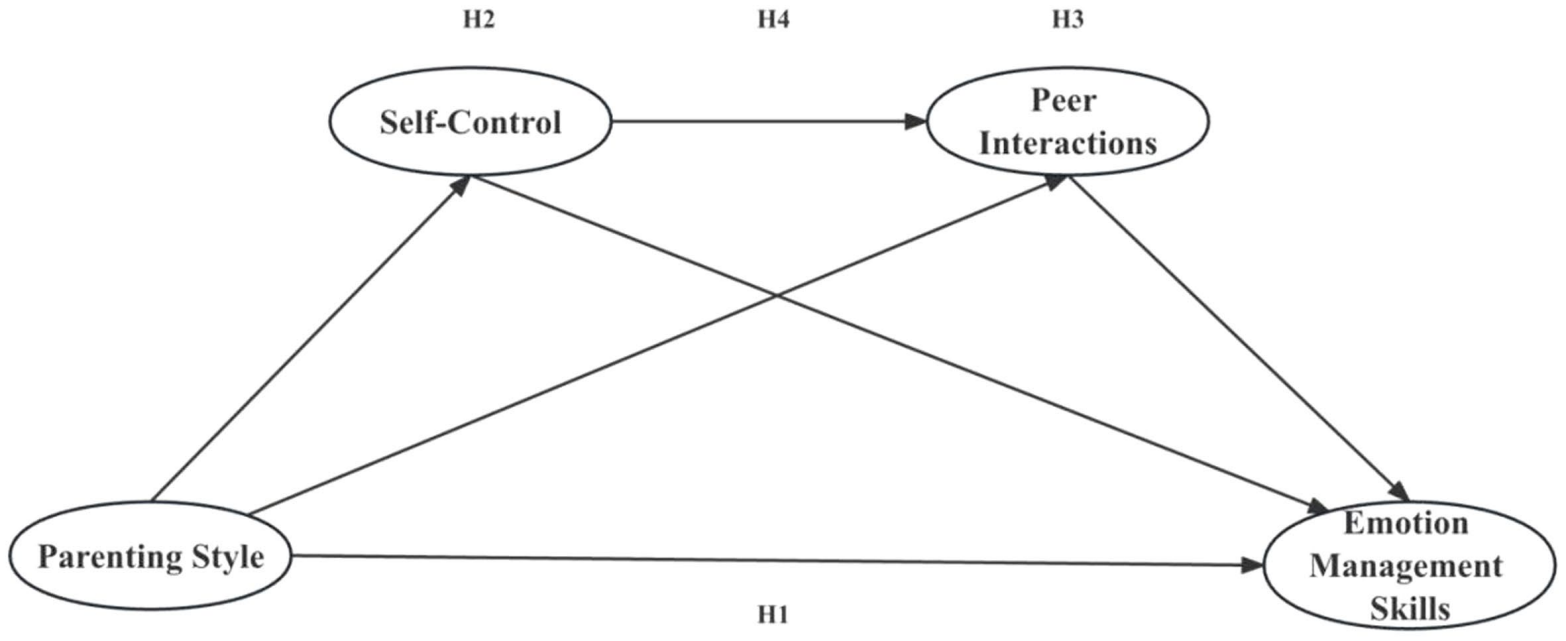
The mediating roles of self-control and peer interactions between authoritarian parenting style and emotion management ability. *p < 0.05, **p < 0.01, ***p < 0.001.

A chain mediation model was tested, which consisted of three indirect effects as follows: (1) self-control played a mediating role in the relationship between parenting styles and children’s emotion management skills; (2) peer interactions mediated the relationship between parenting styles and children’s emotion management skills; and (3) parenting styles may indirectly affect children’s emotion management skills through the chain mediation of self-control and peer interactions (Figure 1).

**Figure 2:**
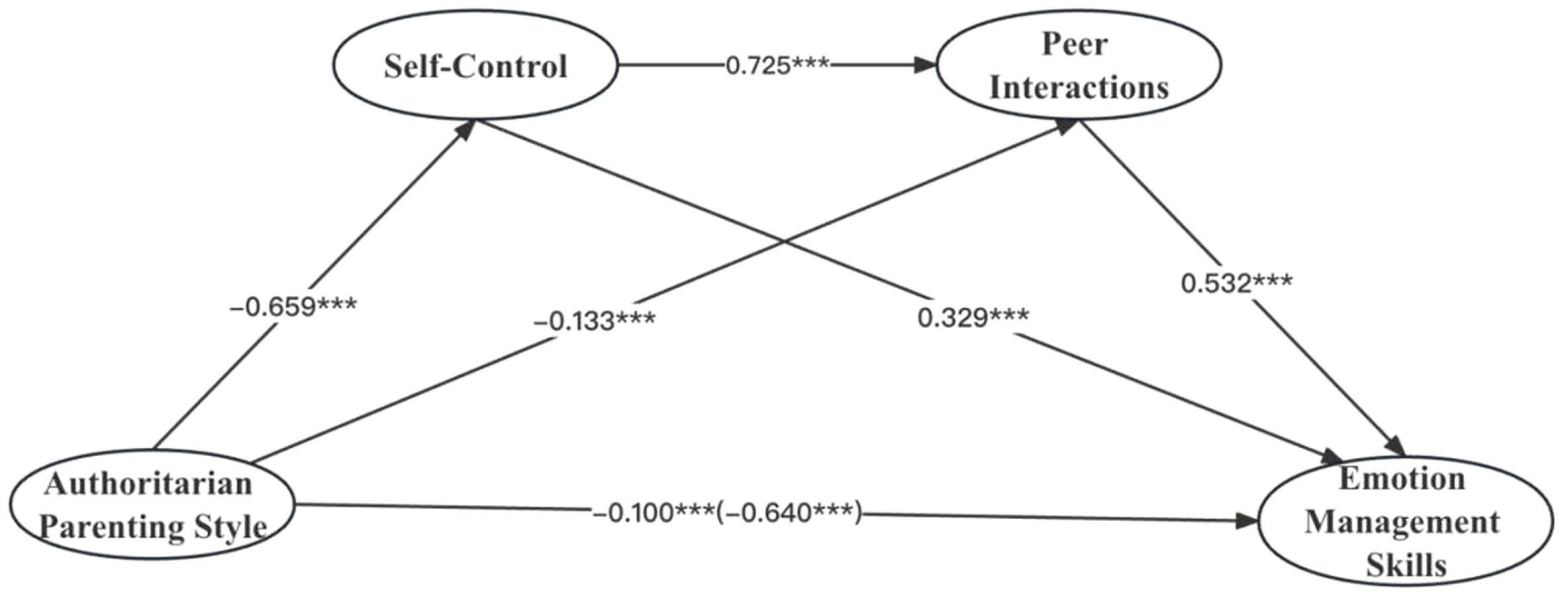
Research hypothesis model.

The SEM was used to examine the mediating effects of self- control and peer interactions between parenting styles and emotion management skills. The results showed that authoritarian parenting style and emotion management skills established significant and negative relationships (*β* = −0.640, *t* = −52.028, *p* < 0.001). After accounting for the influences of control variables, the mediation model showed that authoritarian parenting style had a significant negative direct effect on both self-control (*β* = −0.659, *t* = −49.244, *p* < 0.001) and emotion management skills (*β* = −0.100, *t* = −5.080, *p* < 0.001). Meanwhile, self-control had a significant positive direct effect on both the peer interactions (*β* = 0.725, *t* = 40.725, *p* < 0.001) and emotion management skills (*β* = 0.329, *t* = 12.201, *p* < 0.001). The peer interactions had a significant positive direct effect on emotion management skills (*β* = 0.532, *t* = 122.453, *p* < 0.001; see Figure 2).

**Figure 3:**
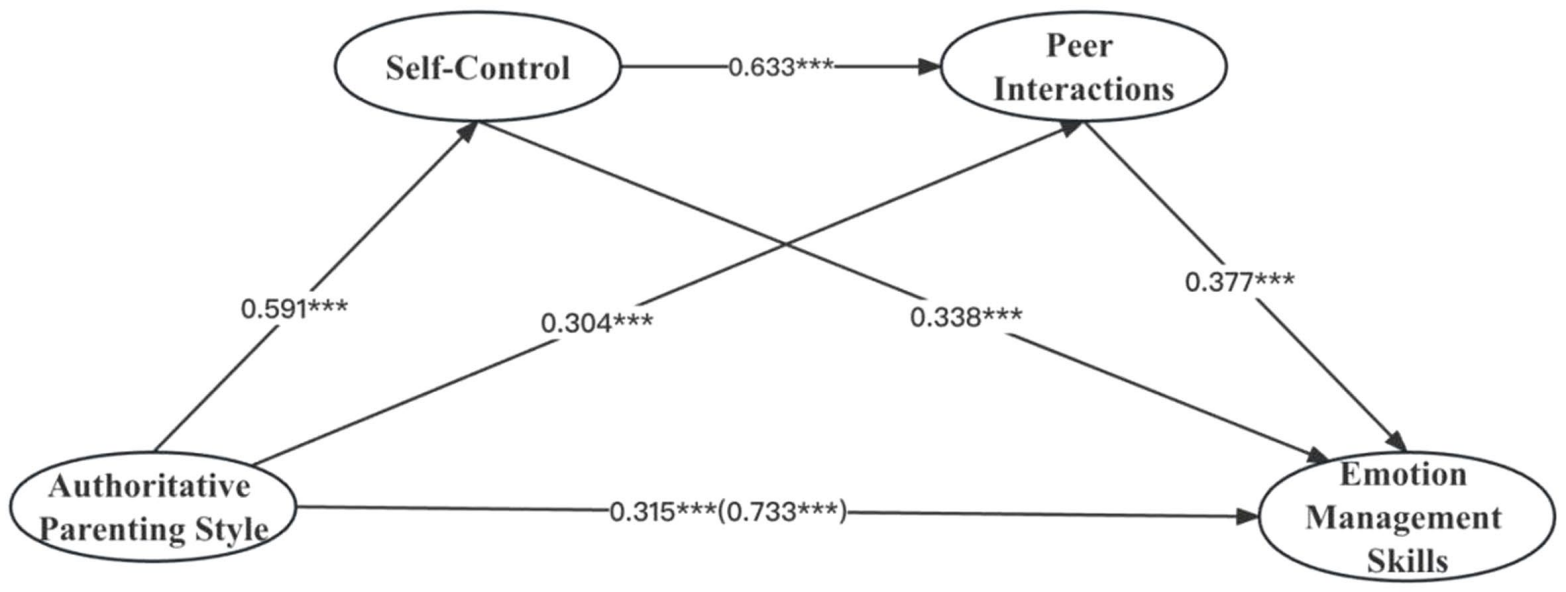
The mediating roles of self-control and peer interactions between authoritative parenting style and emotion management ability. *p < 0.05, **p < 0.01, ***p < 0.001.

As shown in Figure 3, authoritative parenting style had a significant positive direct effect on self-control (*β* = 0.591, *t* = 39.157, *p* < 0.001), peer interactions (*β* = 0.304, *t* = 15.867, *p* < 0.001), and emotion management skills (*β* = 0.315, *t* = 19.139, *p* < 0.001). Self-control had a significant positive direct effect on both peer interactions (*β* = 0.633, *t* = 35.437, *p* < 0.001) and emotion management skills (*β* = 0.338, *t* = 14.527, *p* < 0.001). Peer interactions had a significant positive direct effect on emotion management skills (*β* = 0.377, *t* = 14.988, *p* < 0.001).

Furthermore, as shown in Table 2, the total effect of authoritarian parenting style on emotion management skills was −0.641 (SE = 0.018, 95% CI [−0.674, −0.604], *p* < 0.001) and the direct effect was −0.100 (SE = 0.020, 95% CI [−0.139, −0.062], *p* < 0.001), indicating that both the total effect and the direct effects were statistically significant. The indirect effect was −0.217 (SE = 0.018, 95% CI [−0.254, −0.183], *p* < 0.001) in the pathway of authoritarian parenting style → self-control → emotion management skills, and the mediation effect accounted for 33.801% of the total effect (−0.642). And the indirect effect was −0.071 (SE = 0.010, 95% CI [−0.092, −0.051], *p* < 0.001) in the pathway of authoritarian parenting style → peer interactions → emotion management skills, and the mediation effect accounted for 11.059% of the total effect. Finally, the indirect effect was −0.254(SE = 0.013, 95% CI [−0.280, −0.230], *p* < 0.001) in the pathway of authoritarian parenting style → self-control → peer interactions → emotion management skills, and the mediation effect accounted for 39.564% of the total effect. Then, as shown in Table 3, the total effect of authoritative parenting style on emotion management skills was 0.770 (SE = 0.012, 95% CI [0.747, 0.793], *p* < 0.001), and the direct effect was 0.315 (SE = 0.016, 95% CI [0.282, 0.346], *p* < 0.001), indicating that both the total effect and the direct effect were statistically significant. The indirect effect was 0.200 (SE = 0.014, 95% CI [0.173, 0.230], *p* < 0.001) in the pathway of authoritative parenting style → self-control → emotion management skills, and the mediation effect accounted for 25.940% of the total effect (0.771). And the indirect effect was 0.115 (SE = 0.010, 95% CI [0.095, 0.136], *p* < 0.001) in the pathway of authoritative parenting style → peer interactions → emotion management skills, and the mediation effect accounted for 14.916% of the total effect. Finally, the indirect effect was 0.141 (SE = 0.011, 95% CI [0.121, 0. 163], *p* < 0.001) in the pathway of authoritative parenting style → self-control → peer interactions → emotion management skills, and the mediation effect accounted for 18.288% of the total effect. Given that the bootstrapped 95% confidence intervals do not include zero, the statistical significance of these three indirect effects is established. The data analysis revealed that the indirect effect of parenting styles (authoritarian parenting style and authoritative parenting style) on emotion management skills was contingent upon self-control and peer interactions, which served as significant and positive partial mediators in the association between parenting styles (authoritarian parenting style and authoritative parenting style) and emotion management skills.

**Table 2:** Direct, indirect, and total effects of the hypothesized model.

Model pathways	Estimated effect (*β*)	Boot SE		95% CI	
				Lower	Upper
**Direct effect**					
Authoritarian parenting style → Emotion management skills	−0.100	0.020		−0.139	−0.062
**Indirect effects**					
Authoritarian parenting style → Self-control → Emotion management skills	−0.217***	0.018	33.801%	−0.254	−0.183
Authoritarian parenting style → Peer interactions → Emotion management skills	−0.071***	0.010	11.059%	−0.092	−0.051
Authoritarian parenting style → Self-control → Peer interactions → Emotion management skills	−0.254***	0.013	39.564%	−0.280	−0.230
Total effect	−0.641***	0.018		−0.674	−0.604

Authoritarian parenting style as a predictor variable. *p < 0.05, **p < 0.01, ***p < 0.001.

**Table 3:** Direct, indirect, and total effects of the hypothesized model.

Model pathways	Estimated effect (*β*)	Boot SE	Ration	95% CI	
				Lower	Upper
**Direct effect**					
Authoritative parenting style → Emotion management skills	0.315	0.016		0.282	0.346
**Indirect effects**					
Authoritative parenting style → Self-control → Emotion management skills	0.200***	0.014	25.940%	0.173	0.230
Authoritative parenting style → Peer interactions → Emotion management skills	0.115***	0.010	14.916%	0.095	0.136
Authoritative parenting style → Self-control → Peer interactions → Emotion management skills	0.141***	0.011	18.288%	0.121	0.163
Total effect	0.770***	0.012		0.747	0.793

Authoritative parenting style as a predictor variable. *p < 0.05, **p < 0.01, ***p < 0.001.

## Discussion

This study aimed to investigate the impact of parenting style on children’s emotion management skills, considering the factors of self-control and peer interactions. Our findings validate parental involvement theory (McCurdy and Daro, 2001), bioecological theory (Tudge et al., 2022), and Morris’ triple model of early childhood adjustment (Morris et al., 2017), providing support for the notion that self-control mediates the relationship between parenting style and emotion management skills. Furthermore, peer interactions mediate the relationship between negative parenting style and emotion management skills. Additionally, both self-control and peer interactions serve as chain mediators in the association between parenting style and emotion management skills. Finally, the results of this study support Gottman’s emotion-based approach to parenting and lay the groundwork for determining whether children express constructive or destructive affective tendencies (Jespersen et al., 2021).

First, this study found a significant relationship between parenting style and children’s emotion management skills between the ages of 3–6 (hypothesis one). The evidence confirms that parenting style is the primary factor influencing differences in children’s emotion management skills, and that the more authoritative parenting attitudes and behaviors are, the stronger children’s emotion management skills have, with a positive association. Whereas authoritarian parenting styles negatively affect children’s emotion management skills. The results of this pathway are consistent with previous studies (Tao et al., 2010; Acar et al., 2019; Cantekin and Akduman, 2020; Kashyap et al., 2020). The direct link between parenting style and emotion management skills provides theoretical support for parental involvement theory (Hoover-Dempsey et al., 2005), which states that parents who take an active role tend to listen patiently to their children rather than reprimanding and punishing them, and are more likely to participate in children’s activities and guide children’s emotional perception, expression, and control either directly or subliminally. In such a family environment, children gain a sense of security, increase their self-confidence and independence, and further improve their emotion management skills balanced by their self-control (Guo Y. et al., 2023). High-quality parent–child interactions result in children feeling supported by their families, accumulating positive energy, being more advantaged in complex social situations, having better self-control, and having positive and optimistic stability (Dinkha et al., 2023; Li et al., 2023).

A longitudinal study of parental behaviors related to children in the United States highlighted warmth and emotionally democratic parenting as determinants of good social and emotional development in children (Garthe et al., 2015). However, according to social learning theory, the more often parents adopt authoritarian parenting attitudes that are less supportive and more intrusive, the more difficult it is for children to regulate their emotions during parent–child interaction tasks (NICHD Early Child Care Research Network, 2004). Children tend to hide their emotions or have variable emotions, intimacy is relatively difficult to establish, and the parent–child bonding is in an unhealthy state of development (Hollenstein and Lewis, 2006; Duncan et al., 2009; Tao et al., 2020). The “helicopter parenting” of a collectivist society produces “good children who know what they are doing” and “subordinates who do not think for themselves but follow orders (Ho et al., 2022; Hwang et al., 2023). “Helicopter parents” interfere and manipulate their children’s thoughts and behavior always, and help them decide everything. In this kind of authoritarian family in China, children are only responsible for studying, working and living according to the path planned by their parents or other adults, so there are a large number of “giant babies” with high scores and low abilities who can only study but not actually live (Zhang et al., 2022). Their emotional changes are completely dependent on their parents’ approval, and their emotional stability is brought about by getting used to being planned every step of the way. When they lose the guidance of their parents or teachers, they experience anxiety symptoms of restlessness, worry, confusion, and are in emotional dysregulation and self-doubt (Cui et al., 2019), which seriously affect their physical and mental health (Gao et al., 2023). Based on the above discussion, firstly, in the process of parenting, parents should not only play the role of modeling emotions to improve the quality of parent–child bonding interaction, but also convey positive emotions and emotion management strategies. Secondly, parents should provide guidance and effective communication about children’s hidden and variable emotions, and focus on their inadequate emotion management or behavioral withdrawal, as both may be the cause and effect of children’s behavioral problems.

Second, this study revealed that self-control mediates the relationship between parenting style and children’s emotion management skills, i.e., authoritarian parenting mitigates the negative impact on children’s ability to manage their emotions through greater self-control (hypothesis two). This is similar to findings from previous studies (Özdemir et al., 2013; Briki, 2020; Pan et al., 2021). Overall, the internal mechanisms by which parenting style affect children’s emotion management skills are consistent. As an extension of previous research, the present study shows that parenting style influence children’s emotion management skills through their self-control. This suggests that self-control may be an important factor in protecting against emotional disorders.

During early childhood, the development of self-control is influenced by a range of environmental stressors and supports, shaping its trajectory (Center on the Developing Child at Harvard University, 2011). Individual variations in child self-control have been found to predict outcomes in adulthood, including physical and mental health, criminal behavior, and socioeconomic status (Gagne, 2017). Parenting style plays a significant role in shaping individual differences in self-control among children and adolescents (Gibson et al., 2010; Wang et al., 2016; Li J. B. et al., 2019; Li Y. et al., 2019). On the one hand, with a more stable parent–child relationship, children have a greater sense of integration and trust in the family environment, better control over themselves, and high self-control to better regulate their attentional and behavioral problems so as to display appropriate emotional states (Schmeichel and Baumeister, 2010). Establishing good intimacy between children and parents also promotes mutual emotional contagion and emotional management skills. Therefore, a positive, enthusiastic, and responsive authoritative parenting style can support and promote children’s ability to self-regulate, whereas a negative, harsh, and insensitive authoritarian parenting style appears to have detrimental effects (Gajos and Beaver, 2016). On the other hand, when children’s emotion management skills are not available from the foundational source of parenting style, the ontological factor of self-control comes into play. Research has shown that people with higher levels of self-management tend to have rational self-perceptions and positive self-initiatives (Vohs et al., 2014). Emotional stability and switching between different emotions require the ego to have a more sensitive sense of perception and a stronger level of control. As a result, children’s mastery of self-control will build up their strong willpower from their inner being, promote the development of their good personalities and the successive formation of comprehensive abilities. External emotional management and behavioral dominance will increase in response to more positive evaluations from others, allowing children to integrate more quickly and comfortably into their social or natural environments. At the same time, children’s emotional responses and behaviors become more ethical and promote the development of socialization.

Third, the present study also found a mediating effect of peer interactions between parenting style and emotion management skills, where authoritative parenting style could improve children’s emotion management skills by influencing their peer interactions. This finding is consistent with national and international research that higher levels of peer interactions are associated with authoritative parenting (Bornstein and Bornstein, 2007; Morris et al., 2021; Lanjekar et al., 2022), suggesting that peer interactions play an important mediating role in the way parenting styles influence children’s emotion management skills. Authoritative parenting styles contribute positively to children’s peer interactions in a study of the relationship between effortful control and character anger in Chinese children, and that higher peer interactions enhances children’s good social adjustment function and cultivates individual pro-sociality and a sense of collective responsibility (Zhou et al., 2004). Well-functioning, well-adjusted families have the capacity to successfully mitigate a wide range of developmental threats to children, while at the same time reducing the likelihood of maladjustment in at-risk groups of children (Azimi et al., 2012).

The most significant difference between peer relationships and parent–child and teacher-student relationships is the “equality” of peer groups, which makes it clear that peer interactions are egalitarian and can be constructed by choice, which helps children express their emotions, transfer information, and learn from each other (Reitz et al., 2014; Leung et al., 2021). In this model, children are more active and willing to accept each other’s expressions of opinions and emotions, further developing children’s interpersonal skills. Children’s current and future cognitive development and social adjustment will be negatively affected if they have problems interacting with their peers (Bosacki, 2015). The present study found that authoritative parenting styles positively predicted children’s peer interactions, consistent with previous research (Hong et al., 2021; Marcone et al., 2021). The higher the ability to interact with peers, the better their ability to manage their emotions, which is consistent with Eisenberg’s findings (Eisenberg et al., 2001a). Summary of the above, peer interactions play a partial mediating role in the relationship between parenting style and emotion management skills (hypothesis three). First, as children are exposed to a complex and diverse environment, along with the demands of physiological development, they gradually pay attention to external perceptions and imitate the behaviors and emotional expressions of members of that environment, both consciously and unconsciously. Children with authoritative parents are more emotionally stable, have better peer interactions, have fewer externalizing and implicit problem behaviors, and are more socially adjusted, resulting in higher academic achievement (Kim et al., 2018; Steele and McKinney, 2019). Children who form secure attachments with positive parenting styles are more likely to develop responsive, harmonious peer interactions in kindergarten and elementary school, and these children will have more peer support (Coleman, 2003). Second, children’s peer interactions play a mitigating or ameliorating role in the process of the negative effects of authoritarian parenting styles on children’s emotion management skills, consistent with ecosystem theory (Bronfenbrenner and Morris, 2007). Children in authoritarian parenting styles tend to have social impairments, more academic problems, exhibit higher levels of negative emotions, and experience both explicit and implicit problem behaviors (Ng-Knight et al., 2016; Kim et al., 2018). They are often unable to develop a healthy emotional management system that is conducive to the development of psychological distress in children. When children face unpleasant situations and obstacles in such negative parenting, peers can re-engage their own emotional perceptions through communication or distraction, and their ability to manage emotions will provide time to consider emotional control, enabling children to choose the final emotion presented and promoting the continued occurrence of positive emotions. Such children who are able to use a constructive approach to managing negative emotions also achieve higher social efficacy, reducing their chances of rejection and exclusion by peers (Eisenberg et al., 2001b) and re-establishing better emotion management skills in peer interactions. Therefore, peer interactions are a highly contextualized imitation and learning process in which external and internal emotions are more likely to be displayed. Children discover themselves and others as emotional individuals, develop unconscious emotional imitation and conscious emotional awareness (Izard et al., 2008), become more aware of their emotional problems, and continue to acquire skills for managing their emotions.

Forth, this study revealed a chain mediating effect, with self-control and peer interactions playing intermediary roles between parenting style and emotion management skills (hypothesis four). These findings support the idea that children with authoritative parenting styles tend to exhibit higher levels of self-control and engage in more positive peer interactions, thereby facilitating the development of their emotion management skills (Chase-Lansdale et al., 1995; Pan et al., 2021; Papadopoulos, 2021). Conversely, children with authoritarian parenting styles tend to have lower levels of self-control and limited peer interactions, impeding the development of their emotion management skills and leading to emotional dysregulation and behavioral problems. These results are consistent with prior research findings (De Minzi, 2007; Gagnon et al., 2014). According to the situational developmental perspective, the family, community, and culture significantly influence children’s emotional stability, while their attitudes, judgments, emotional perceptions, and behavioral expressions are highly susceptible to external influences from others’ behaviors and group norms (Lamm et al., 2018). Inhibitory control skills are crucial for successful school adjustment, positive peer relationships, and social–emotional functioning (Rueda et al., 2005; Bell and Deater-Deckard, 2007; Crandall et al., 2015). Both inhibitory control and effortful control are important aspects of self-control in children (Scholtes et al., 2021). Given that weaker self-control is associated with lower academic achievement, difficulties in establishing positive peer relationships, and deficits in social–emotional functioning, further research should investigate the ways in which interactive coordination can support healthy developmental outcomes for children (Blair, 2002).

Children who are guided by their parents’ positive emotional concepts and emotional behaviors at an early age will develop better self-control. Simultaneously, children who interact with their peers will develop more positive emotions about themselves and others to create happy atmosphere, and such positive emotions will infect the entire peer group from individual to individual, influencing each other emotion management skills for better or worse. Children with high levels of emotion management have better empathy and are able to put themselves in the shoes of their peers, which not only enhances children’s observational and analytical skills, but also motivates them to share emotional feelings, speculate on emotion-generating motivations, and improve self-social adaptability, consistent with previous research on similar models (Llorca-Mestre et al., 2017). Overall, this finding extends previous research that has examined the complex relationships among parenting style, self-control, peer interactions, and children’s emotion management skills within the framework of Morris’ three-model of early childhood adjustment. Authoritative parenting styles help children develop better self-control and become popular in peer interactions, both of which promote children’s ability to manage their emotions. Conversely, children in authoritarian parenting styles develop poorer levels of self-control and peer interactions that hinder the development of their emotion management skills. This highlights the need to pay more attention to family education and parenting style choices to improve children’s emotional management and mental health, then to shape physically and mentally healthy children through better self-control and peer interactions.

## Implications

This study examined the influence of parenting styles on children’s emotion management skills from the perspective of self-control and peer interactions, and provided theoretical and practical guidance on how families and schools can actively and rationally promote children’s emotion management skills. At the same time, it established a reference direction for parents to choose which parenting styles to implement. Our findings showed that self-control plays an important role in the influence of parenting style on children’s emotion management skills. Parents should promote their children’s self-control by using parenting behaviors that are high in support and understanding, low in rejection and over-interference. Our findings also indicated that peer interactions play an important role in the influence of parenting style on children’s emotion management skills and affect their personality development. Therefore, parents should pay attention not only to the establishment of appropriate self-control but also to the development of their children’s peer interactions. Parents need to cultivate their children’s self-control and peer interactions through parenting attitudes that are high in emotional warmth, high in supportive acceptance, low in rejection, and low in excessive control, which, in turn, promote the growth of their children’s emotion management skills. Specifically, parents must first be sensitive to children’s emotional changes and provide positive emotional experiences to satisfy children’s emotional needs and psychological demands. For example, in their relationship with their children, parents are not only listeners but also companions. They need to excel at observing and guiding their children’s emotional responses and fostering their awareness of emotion management. When parents listen attentively and provide warmth, acceptance, and emotional support, children will perceive more positive emotional feedback from their caregivers and demonstrate stronger emotional skills (Feng et al., 2021). Second, children will learn and regulate uncomfortable emotions in peer interactions, redirecting emotional attention, reducing both the intensity and maintenance of negative emotions, and maintaining emotional health without stressing the mind and body (Li J. B. et al., 2019; Li Y. et al., 2019). Parents and peers play a crucial role in children’s emotional growth and development. Thus, emotional harmony within the family and positive peer relationships are essential for healthy physical and mental development in childhood. This is why early childhood educators should emphasize family education and the cultivation of beneficial peer relationships for children. Ultimately, the combined strengths of the family, preschool, and community create opportunities for children to develop healthy emotion management skills by taking full advantage of multiple environmental resources to learn and experience.

## Limitations and future work

The results of the current study should be considered in light of its limitations. First, due to the cross-sectional design of the data, we could not infer causal relationships among the variables. For example, it is not clear that children’s lower emotional competence is caused by negative parenting or poor peer interactions, a longitudinal study would be needed to explore these possibilities. Second, we controlled for the age factor of children aged 3–6 years in the study, and all variables were reported using other-rated questionnaires. Although multi-subject reports increase the validity of the data, they lack data on children’s self-perceptions, so future studies suggest that researchers can collect data directly from children’s responses and performance through play-based tests combined with other-rated questionnaires to improve measurement accuracy. Third, this study only explored the mediating mechanism of parenting style on children’s emotion management skills without considering individual differences, and future studies could conduct a more in-depth analysis of the relationship between parenting style and emotion management skills from the perspective of regulation mechanism. Fourth, considering the Chinese cultural context in which parental socialization occurs, we focused on comparing and analyzing the two dominant parenting styles, without considering the permissive style from the three-style model. While we have observed the benefits of the demanding and responsive authoritative parenting styles for children’s development, it might be worthwhile to explore the potential advantages of the authoritarian parenting style for children in future research. Fifth, our subjects were selected from China, and culture is also one of the important contextual factors. For example, as a collectivist country, China will show higher collective will and more harmonious consistency (Marginson and Yang, 2020). Therefore, cultural and geographical differences should be considered in the subjects, and the relationship between the variables discussed in the model may be different. Therefore, a larger sample is needed in future studies to build research models that can be generalized to different countries or regions.

## Conclusion

The present research investigated the status of emotion management skills among Chinese children aged 3–6 years, while exploring various preceding factors that exert influence on emotion management and emotional stability. Specifically, this study reveals the differential effects of two different types of parenting styles on children’s emotion management skills. Furthermore, self-control and peer interactions were identified as a potential mediator in the relationship between parenting style and children’s emotion management skills. In addition, this study examined the following mediating pathways: authoritarian parenting style → self-control → peer interactions → emotion management skills and authoritative parenting style → self-control → peer interactions → emotion management skills. As children’s levels of self-control and peer interactions increase, the impact of parenting style on children’s emotion management skills becomes more pronounced. This study contributes both theoretically and practically. Firstly, it enhances the existing theoretical framework concerning the connection between parenting style and children’s emotion management skills, shedding light on the underlying mechanisms by which parenting style impacts the occurrence of children’s emotional well-being. Secondly, this study integrates environmental and individual perspectives in the exploration of potential factors that can ameliorate children’s emotion management skills, which will provide a more comprehensive picture of the development of emotion management in Chinese children between the ages of 3 and 6 years. Lastly, the results of this study provide practical implications for parents seeking the best parenting style to develop good emotion management skills in children and contribute to family education.

## Data availability statement

The raw data supporting the conclusions of this article will be made available by the authors, without undue reservation.

## Ethics statement

The studies involving humans were approved by the Ethics Committee Review Board of Liaoning Normal University. The studies were conducted in accordance with the local legislation and institutional requirements. The participants provided their written informed consent to participate in this study. Written informed consent was obtained from the individual(s) for the publication of any potentially identifiable images or data included in this article.

## Author contributions

DL: writing—original draft, writing—review and editing. WL: conceptualization, methodology, software, formal analysis, writing—original draft, writing—review and editing. XZ: writing—original draft, writing—review and editing. All authors contributed to the article and approved the submitted version.

## Funding

This research was funded by the Foundation of Liaoning Province Education Administration‘s Key Research Project for Universities, grant number LJKZR20220114.

## Conflict of interest

The authors declare that the research was conducted in the absence of any commercial or financial relationships that could be construed as a potential conflict of interest.

## Publisher’s note

All claims expressed in this article are solely those of the authors and do not necessarily represent those of their affiliated organizations, or those of the publisher, the editors and the reviewers. Any product that may be evaluated in this article, or claim that may be made by its manufacturer, is not guaranteed or endorsed by the publisher.
